# The TIMELESS effort for timely DNA replication and protection

**DOI:** 10.1007/s00018-023-04738-3

**Published:** 2023-03-09

**Authors:** Jinal A. Patel, Hyungjin Kim

**Affiliations:** 1 Department of Pharmacological Sciences, State University of New York at Stony Brook, Basic Sciences Tower 8-125, 101 Nicolls Rd, Stony Brook, NY 11794 USA; 2 Stony Brook Cancer Center and Renaissance School of Medicine, Stony Brook University, Basic Sciences Tower 8-125, 101 Nicolls Rd, Stony Brook, NY 11794 USA

**Keywords:** DNA replication, Fork protection complex, Replisome, Genome stability, ATR-CHK1 checkpoint

## Abstract

Accurate replication of the genome is fundamental to cellular survival and tumor prevention. The DNA replication fork is vulnerable to DNA lesions and damages that impair replisome progression, and improper control over DNA replication stress inevitably causes fork stalling and collapse, a major source of genome instability that fuels tumorigenesis. The integrity of the DNA replication fork is maintained by the fork protection complex (FPC), in which TIMELESS (TIM) constitutes a key scaffold that couples the CMG helicase and replicative polymerase activities, in conjunction with its interaction with other proteins associated with the replication machinery. Loss of TIM or the FPC in general results in impaired fork progression, elevated fork stalling and breakage, and a defect in replication checkpoint activation, thus underscoring its pivotal role in protecting the integrity of both active and stalled replication forks. TIM is upregulated in multiple cancers, which may represent a replication vulnerability of cancer cells that could be exploited for new therapies. Here, we discuss recent advances on our understanding of the multifaceted roles of TIM in DNA replication and stalled fork protection, and how its complex functions are engaged in collaboration with other genome surveillance and maintenance factors.

## Introduction

DNA replication is the most vital biological process for an organism, and its defects in DNA replication and repair are associated with many disorders, including cancer. DNA replication fork integrity is primarily preserved by the fork protection complex (FPC), which consists of TIMELESS (TIM) and its obligate heterodimeric partner TIPIN (Swi1 and Swi3 in *Saccharomyces pombe*, and Tof1 and Csm3 in *Saccharomyces cerevisiae*), along with CLASPIN/Mrc1 and AND-1/Ctf4 [1]. The FPC forms a scaffold for the replisome to ensure unperturbed fork progression and acts as a mediator of the DNA replication checkpoint at stalled forks. Recent studies have identified SDE2 and its single-stranded DNA (ssDNA) binding property via the SAP (SAF-A/B, Acinus, PIAS) domain as a regulatory element of the FPC to stabilize the TIM protein and its localization at replication forks, thereby supporting replisome activity [2, 3]. The nomenclature of “*timeless*” originated from *Drosophila* TIM (dTIM), which, in complex with PERIOD (PER), acts as a key regulator of the animal circadian clock powered by transcription–translation feedback loops [4]. However, mammalian TIM (mTIM) turned out to be an ortholog of dTIMEOUT or dTIM2, which unlike dTIM, genetically interacts with ATR (ataxia-telangiectasia and Rad3-related kinase) and is essential for the maintenance of chromosome integrity [5]. Consistent with this notion, over a decade of structural, biochemical, and cellular studies have revealed the critical non-circadian roles of mTIM as well as Swi1 and Tof1 from yeast in controlling DNA replication as a replication-fork-associated factor and collaborating with DNA damage response pathways to protect DNA replication fork integrity against endogenous and exogenous genotoxic assaults. We herein review the collective functions of TIM in the context of the FPC in coordinating diverse genome maintenance processes at the replication fork as well as its impact in tumorigenesis.

## The structure of the fork protection complex

**Fig. 1:**
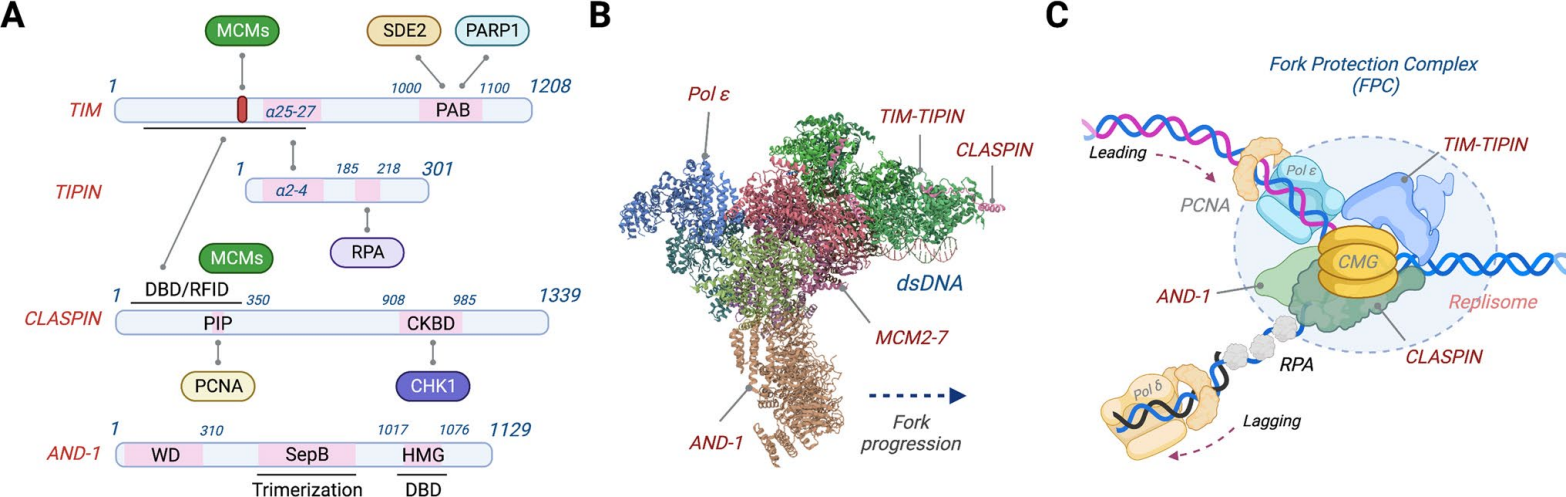
The structure of the fork protection complex at DNA replication forks. **A** Schematic describing the constituents of the human fork protection complex (FPC) and its interacting proteins. TIMELESS (TIM) directly interacts with TIPIN via its α-helices 25–27, which sits onto a hydrophobic interface formed by the α-helical helix–turn–helix (HTH) domain 2–4 of TIPIN (helix numbers from [10]). In the absence of each other, the hydrophobic interface becomes exposed, which results in the instability of TIM or TIPIN. The TIM MCM-plugin (red box) mediates the association of the TIM–TIPIN complex with the N-terminal portion of the MCM2-7 complex. The C-terminus of TIM directly interacts with both PARP1 via a PARP1-binding (PAB) domain and SDE2, a regulatory protein that stabilizes TIM. The C-terminus of TIPIN exhibits a conserved RPA-binding motif, which directly interacts with RPA32. The N-terminus of CLASPIN constitutes a DNA-binding domain (DBD) or replication fork-interacting domain (RFID) necessary for its association with chromatin and binding to branched or forked DNA in vitro [103]. This domain also mediates the interaction of CLASPIN with TIM, which not only encompasses the regions that make extensive contacts with MCM2-7 as part of the replisome but also harbors a PCNA-interacting protein (PIP) motif [10, 32]. The C-terminus of CLASPIN displays a CHK1 kinase binding domain (CKBD) necessary for the interaction with CHK1 during checkpoint activation (referred from a *Xenopus* CKBD defined by amino acids 847–903) [102]. AND-1 exhibits N-terminal WD repeats necessary for its protein–protein interactions, a SepB domain for AND-1 trimerization in the middle region, and an HMG-box at the C-terminus required for DNA interaction. **B** A cryo-EM structure of the core human replisome, visualizing the CMG, Pol ε, TIM–TIPIN, CLASPIN, and AND-1 bound to a DNA replication fork (adapted from PDB: 7PFO and processed by Biorender). The structure of CLASPIN was not fully resolved, exhibiting only a fraction of the polypeptide. **C** The architecture of a DNA replication fork supported by the FPC. The CMG helicase complex unwinds the DNA duplex, while replicative DNA polymerases ε and δ incorporate DNA nucleotides to leading and lagging strands, respectively, guided by PCNA as a processivity factor for the replicative polymerase progression. The TIM–TIPIN heterodimer cradles the DNA duplex in front of the CMG helicase–polymerase complex, stabilizing its association at a DNA replication fork and facilitating strand separation. Its occupancy at the front of CMG may also restrict the replisome activity to promote replisome pausing when encountering difficult-to-replicate DNA regions or under genotoxic stress. The cryo-EM structure implies that CLASPIN stretches across the replisome, making an extended contact with TIM at the front and the MCM subunits/CDC45 (not shown) at the back, thereby coordinating template unwinding and pol ε-mediated leading-strand synthesis [10]. The AND-1 trimer forms an interface with CMG and DNA at the back of the replication fork

TIM exhibits an evolutionarily conserved interaction with its obligate binding partner TIPIN, and thus the cellular stability of TIM and TIPIN is interdependent (Fig. 1A) [6]. Initially, the X-ray crystal structure of the TIM-TIPIN complex defined the N-terminal segment of human TIM in interacting with TIPIN [7], but the updated structure from *C. thermophilum* Tof1–Csm3 complex revealed that Csm3 binds to the middle region of Tof1 and that the complex together forms a single extended α-helical repeat fold, in which Csm3 caps on the C-terminal end of Tof1 that consists of eight armadillo repeats [8]. The four helix–turn–helix domains of Csm3 form a hydrophobic core, which accommodates a hydrophobic helix present at the C-terminal end of Tof1, providing an explanation for the mutual dependence on their stability and highlighting the scaffold role in supporting the replisome. Recent cryo-electron microscopy (cryo-EM) structural analyses of over 1.4 MDa yeast and human replisomes also revealed similar features of the TIM–TIPIN interaction and, at the same time, have allowed us to visualize the overall architecture of the FPC within the replisome (Fig. 1B) [9, 10]. Surprisingly, the TIM–TIPIN complex interacts with the leading edge or N-tier of the CMG (CDC45, MCM2-7, GINS) helicase complex and forms a positively charged groove that is positioned ahead of the CMG to grip and stabilize the parental duplex DNA, which facilitates strand separation by the MCM2-7 core. This indicates that the TIM–TIPIN complex does not physically tether the CMG and replicative polymerases but rather prevents unrestrained replisome uncoupling via its occupancy at the front of a replication fork (Fig. 1C). While CLASPIN/Mrc1 does not alter the overall structure of the replisome, it embraces the replisome in an extensive and flexible manner, in which its N-terminus interacts with TIM and directly contacts the duplex DNA, while its interaction stretches across CDC45 and MCM subunits [9, 10]. Its ability to interact with many components across the replisome suggests that CLASPIN may coordinate template unwinding and leading-strand progression, which together with the TIM–TIPIN complex controls rates of DNA replication and ensures replisome coupling in order to limit ssDNA exposure. Indeed, similar to TIM, CLAPSIN is necessary for efficient fork progression in both yeast and human cells [11–14]. Finally, AND-1/Ctf4 is involved in DNA replication by bridging DNA polymerase α (Pol α) and the CMG at DNA replication forks, and at least in vertebrates, its interaction with DNA pol α/primase is mediated by the C-terminal high-mobility group (HMG) box that is uniquely present in AND-1 [15, 16]. Crystallographic analyses revealed that the yeast Ctf4 forms a trimer, which not only links the CMG and pol α but also acts as a hub to recruit multiple ancillary proteins that harbor a Ctf4-interacting-peptide (CIP) box to the CMG [17, 18]. This includes the Dna2 nuclease and the rDNA-associated protein Tof2, indicating that AND-1/Ctf4 may help the FPC coordinate with other cellular processes such as rDNA copy-number regulation to DNA synthesis [18]. In the future, biochemical and functional characterization of key residues necessary for the protein–protein and DNA–protein interactions identified in these structures will provide new mechanistic insight into how the FPC is engaged to a replication fork to control the replisome activity.

## Biochemical properties of the fork protection complex

Biochemical studies using the purified components of TIM and other FPC proteins have provided valuable insight into how the replisome activity is regulated at ongoing replication forks. Consistent with recent structural data, an early study with the baculovirus-derived recombinant TIM protein revealed that TIM stably interacts with the CMG but not with replicative polymerases in vitro [19]. Interestingly, TIM inhibits DNA-dependent ATPase and DNA unwinding activities of the multiple MCM protein complexes, while it stimulates the activities of DNA polymerases α, δ, and ε, suggesting that TIM directly affects the catalytic function of the replisome [19, 20]. Therefore, the TIM–TIPIN complex may act as a negative regulator of the CMG that constrains unrestricted DNA unwinding activity away from nascent DNA synthesis, which is in line with the results from cryo-EM studies that demonstrate its role in supporting the replisome activity in front of the replisome [9, 10]. At unchallenged forks, impaired fork progression noted in TIM-deficient cells may partly result from a lack of polymerase stimulation where the processivity activity would drive DNA unwinding. On the other hand, under conditions of replication damage, restricting DNA unwinding activity ahead of the replication fork may be necessary for allowing the replisome to pause at stalled forks and thus preventing the uncoupling of helicase–polymerase activities. Recent reconstitution of the *S. cerevisiae* replisome proteins in vitro provided more direct evidence on the catalytic regulation of the replisome by the FPC. While the minimally reconstituted replisome turned out to synthesize DNA very slowly, addition of purified Mrc1–Tof1–Csm3 proteins significantly accelerated the rate of replisome progression on DNA, presumably by stimulating the rate of template unwinding by the CMG [21, 22]. Interestingly, Mrc1 is able to stimulate fork progression without Tof1–Csm3 but not vice versa, indicating that the Tof1–Csm3 complex augments the function of Mrc1. An additional single-molecule imaging study revealed that the Mrc1-Tof1-Csm3 complex enhances the rate of leading-strand replisome progression in a dynamic and transient fashion, implicating its role in alternating phases of variable fork speeds [23]. The N-terminus of Tof1 interacts with Mrc1, suggesting that Tof1 may be necessary for the stable association of Mrc1 at replication forks as revealed by co-immunoprecipitation (IP) and chromatin IP (ChIP)-on-chip analyses [24]. Furthermore, the N-terminal portion of Mrc1 interacts with both the CMG and pol ε, suggesting that tethering of both the front and back of the replisome by Mrc1 may contribute to the helicase–polymerase coupling, and thus allowing for efficient fork progression [9, 25, 26]. Similar results were observed with purified human TIM–TIPIN and CLASPIN proteins, in which CLASPIN stimulates leading-strand replication with the help of TIM–TIPIN [27]. In this in vitro system, the direct interaction between TIM and CLASPIN was required for maximum fork progression activity, further corroborating the idea that one important role of TIM is to stably position CLASPIN and support its activity at the replication fork. While the overall structure of yeast and human core replisomes is remarkably similar, human AND-1 participates in augmenting leading-strand progression presumably via its C-terminal HMG DNA-binding domain, which is not present in yeast Ctf4 [16]. Together, a series of biochemical studies suggest that TIM–TIPIN (Tof1–Csm3) promotes efficient fork progression by providing a structural scaffold of the replisome. TIM stabilizes CLASPIN association with the replication fork and augments its replication-promoting function. Additionally, localization of TIM at the front of the replisome may enable TIM to restrict CMG unwinding activity, and thus fine-tune replication fork progression, which would become particularly important for regulating the replisome in response to fork barriers and replication damage (as discussed below).

## Roles of TIMELESS in DNA replication

### Replication fork progression and pausing

In both yeast and mammals, TIM in the FPC is a replication fork-associated factor that is recruited to replication origins at the onset of S-phase and travels with the replication machinery [28–30]. TIM depletion in human cell lines leads to aberrant accumulation of the CMG onto the chromatin. Nevertheless, this abnormal CMG complex is not properly phosphorylated by the CDC7–Dbf4 kinase, thus resulting in reduced DNA unwinding activity, suggesting that TIM is required for the proper chromatin association of the CMG for timely S-phase progression [31]. Additionally, CLASPIN is known to recruit CDC7 for efficient phosphorylation of MCM proteins, which is required for the initiation of DNA replication [32]. siRNA-mediated knockdown of TIM or deletion of Tof1 results in shorter replication tracks as measured by DNA fiber analysis, indicating that TIM is necessary for efficient fork progression [2, 14, 33, 34]. This is accompanied by an increase in the number of asymmetric forks, an indication of frequent fork stalling, and a decrease in inter-origin distance, representing compensatory origin firing to alleviate replication fork damage in the absence of TIM [2].

While promotion of replisome processivity is a key function of the FPC, the TIM–TIPIN complex is also necessary for transient fork pausing at various fork barriers encountered during DNA replication. The paused fork maintains the overall integrity of the replisome, in which Tof1–Csm3, but not Mrc1, Mec1 (ATR), or Rad53 (CHK1/CHK2), is necessary for mediating pausing at non-nucleosomal proteins bound to DNA [35]. In response to hydroxyurea (HU), which depletes cellular deoxynucleotide (dNTP) pools, Tof1 or Mrc1 mutants exhibit a temporal increase in fork velocity and uncoupling of Cdc45 from DNA synthesis activity, which is associated with ssDNA accumulation and activation of the S-phase checkpoint [28]. This indicates that the FPC constitutes a ‘replication-pausing complex’ that keeps DNA replication in check and thus limits deleterious consequences to the fork structure under replication stress. Indeed, Swi1 deletion leads to fork collapse and DNA rearrangements in ribosomal DNA (rDNA) repeats, marked by an increase in Rad22 (RAD52) foci and the formation of Holliday junction-like structures [30, 36]. Similarly, Tim deficiency in mouse embryonic fibroblasts increases sister chromatid exchange that is dependent on BRCA2/RAD51-mediated homologous recombination (HR), further highlighting the role of TIM in suppressing genome instability throughout S-phase by ensuring unperturbed DNA replication and preventing the formation of aberrant chromosome structures [37].

### Proteinaceous fork barriers

**Fig. 2:**
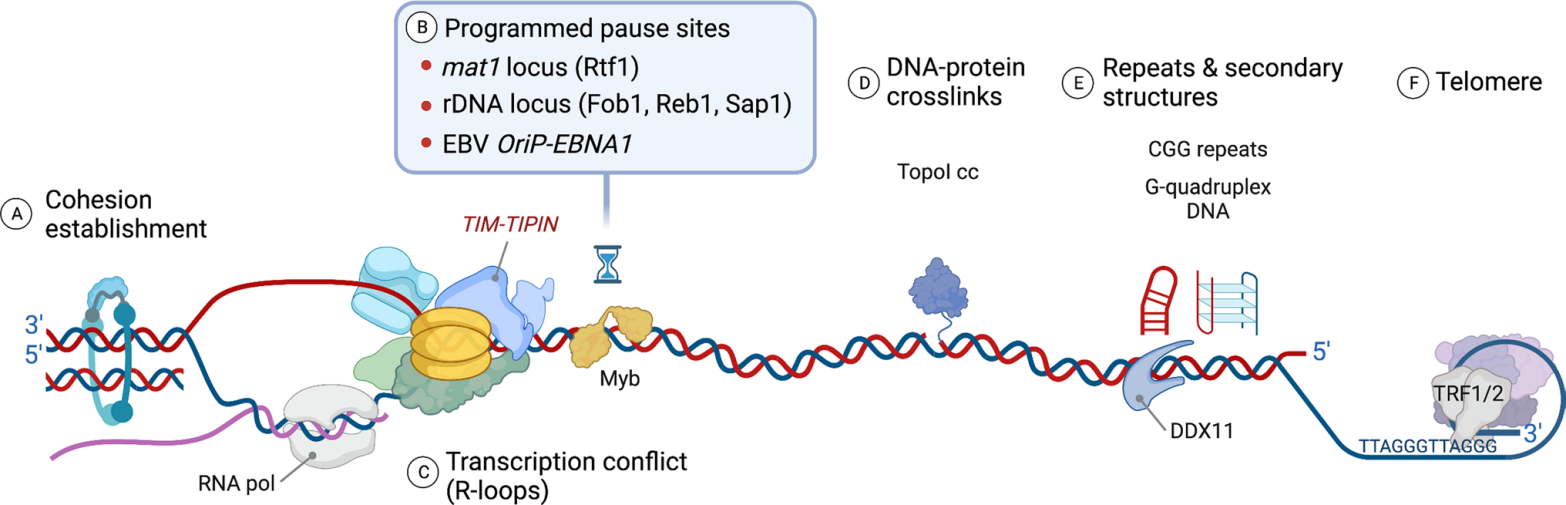
Regulation of replication fork progression and pausing by the fork protection complex. TIM in the fork protection complex not only promotes replication fork progression by coupling the CMG helicase and replicative polymerases, but also pauses replisome progression to handle DNA regions that present obstacles for fork elongation. **A** Besides its role in stabilizing the replisome, TIM is required for the establishment and association of the cohesin complex to ensure proper pairing and segregation of chromosomes, indicating that TIM coordinates DNA synthesis with the sister chromatid cohesion process. **B** TIM is necessary for pausing at ‘programmed pausing sites’, marked by DNA–protein interacting barriers, in which a replication fork needs to pause transiently to control the direction of fork progression and induce polar fork arrest while navigating through fork blocking sites. These include the *S. pombe* mating-type (*mat1*) locus RTS1 bounded by a Myb factor Rtf1, the ribosomal DNA (rDNA) locus bound by Fob1 in *S. cerevisiae*, and the FPC-dependent rDNA polar fork barriers bound by Reb1 and Sap1 in *S. pombe*. TIM is also required for pausing and termination at *oriP* of the Epstein–Barr virus (EBV) episomal DNA. **C** The transcription machinery causes transcription–replication conflicts by colliding with the replisome in the head-on or co-directional orientation, presenting a protein barrier that impedes fork progression. Unresolved DNA–RNA hybrids, or R-loops, also induce fork stalling, DNA recombination, and breakage, posing a threat to genome integrity. **D** Proteinaceous replication fork barriers are often covalently attached to the DNA backbone, for instance topoisomerase I cleavage complexes (TopoI cc), and require TIM for pausing at the front of the DNA–protein crosslinks (DPC) and resolving them. Recruitment of Topoisomerases to the replisome by TIM ensures the relief of torsional stress ahead of the CMG helicase and prevent excessive DNA precatenation. **E** TIM minimizes fork stalling and facilitates fork movement through intrinsically difficult-to-replicate chromosome regions such as repetitive DNA and secondary DNA structures (e.g., G-quadruplexes). TIM-associated helicases such as DDX11/ChlR1 may coordinate with the fork protection complex to resolve DNA structure-related fork barriers and travel through cohesion sites. **F** TIM is necessary for telomere replication, in which the replication fork encounters both DNA-bound protection complexes (e.g., TRF1/2 in the shelterin complex) and repetitive G-rich telomeric sequences

The TIM–TIPIN complex placed at the front of the replisome is ideally suited to recognize and respond to various obstacles encountered during fork progression. Specifically, TIM is required for progressing through distinct barriers that impede DNA replication, which often requires timely pausing and subsequent passage over difficult-to-replicate regions of DNA (Fig. 2). These include DNA replication–transcription conflict sites, programmed pausing sites marked by protein–DNA interacting barriers, and repetitive sequences that exhibit distinct secondary DNA structures. For instance, replication forks from Swi1 or Swi3 mutants in *S. pombe* fail to pause at the genetically programed pause sites near the mating-type (*mat1*) locus, namely the *mat1* replication pause site 1 (MPS1) and the replication termination site 1 (RTS1), resulting in a defect in mating-type switching [38]. Similarly, Tof1–Csm3 in *S. cerevisiae* is necessary for pausing at the rDNA locus, which contains a replication fork barrier (RFB) bound by a site-specific replication terminator protein, Fob1, by protecting Fob1 from being displaced by the Rrm3 helicase [39]. Recently, fork pausing events at the protein-bound DNA sites were reconstituted in vitro, where Tof1–Csm3 and Fob1 were shown to be necessary and sufficient for pausing at replication fork barriers in an orientation-dependent manner [40]. Tof1–Csm3 also promotes fork pausing independently of Rrm3, which involves recruitment of topoisomerase I (TopoI) to the replication fork to relieve positive torsional stress ahead of the replisome [41]. Interestingly, another way to relax topological stress is fork rotation at the expense of generating DNA precatenanes, newly replicated interwined DNA duplexes that form behind the fork [42]. It was shown that Tof1–Csm3 restricts fork rotation in the context of difficult-to-replicate sites, such as irreversible TopoI–DNA crosslinks formed ahead of the fork and DNA replication termination sites [43]. Accordingly, excessive fork rotation and precatenation is observed in the absence of Tof1–Csm3, which causes replication-associated DNA damage and chromosome fragility, perhaps by the loss of resistance to fork rotation via overall replisome disintegration and/or the inability to properly localize TopoI at the fork and relieve torsional stress.

In *S. pombe*, Swi1–Swi3 acts on protein–DNA replication fork barriers at three termini sites (*Ter 1, 2,* and *3*) in the spacer regions of rDNAs to promote polar fork arrest, and thus prevent replication–transcription conflicts [44, 45]. Likewise, TIM in human cells is necessary for polar fork arrest at the transcription terminator Sal box T1, which binds to polymerase I transcription termination factor 1 (TTF-1), highlighting the conserved role of TIM in coordinating replication and transcription activity in highly transcribed rDNA genes [46]. Interestingly, the ability to pause DNA replication forks at various protein–DNA barriers during unchallenged S-phase progression seems to be specific to Tof1, but not Mrc1 [11]. This reinforces the notion that Tof1 may act as a ‘brake’ to slow down the progression of the CMG when it encounters certain protein barriers, while Mrc1 primarily governs the rate of fork progression, consistent with structural and biochemical analyses of the FPC described above. Similar functions may operate at stalled forks caused by genotoxins and DNA lesions, but ATR–CHK1 signaling may play distinct roles in regulating TIM and CLASPIN for pausing the replisome and stabilizing stalled forks as well. Intriguingly, the host TIM–TIPIN is also exploited to support the replication and maintenance of the Epstein–Barr virus (EBV) episomal DNA by facilitating pausing and termination at the origin of plasmid replication (*oriP*) region [47]. This repetitive DNA recognition element was shown to be transiently crosslinked by the EBV nuclear antigen 1 (EBNA1), one of the latent proteins essential for EBV long-term persistence and oncogenic transformation, to promote replication termination and viral episome maintenance, further underscoring the role of TIM in pausing of the replisome at protein barriers, including covalent DNA–protein adducts [48].

### DNA repeats and secondary structures

Instead of enforcing replication fork pausing at protein barriers, TIM also plays a key role in navigating through intrinsically difficult-to-replicate DNA structures by counteracting replication fork stalling while promoting replisome progression. Fork stalling at CGG repeats in the 5′ untranslated region of the *FMR1* gene frequently leads to expansion of repeat sequences and formation of secondary structures, resulting in fork breakage and genetic disorders such as Fragile-X syndrome [49]. Tof1 and Mrc1 are required for facilitating replication fork progression through CGG repeats and suppressing the expansion of these repeats [50]. Similarly, Tof1 deficiency leads to a large-scale expansion of Friedreich’s ataxia GAA repeats in the *S. cerevisiae* genome [51]. In human cells, depletion of TIM, TIPIN, or CLASPIN greatly accelerates CTG(n)·CAG(n) repeat expansion, thus resulting in the chromosomal instability associated with myotonic dystrophy type 1 (DM1) [52]. While both Tof1 and Mrc1 are required for preventing the instability of long CAG repeats, the checkpoint function of Mrc1 seems dispensable, indicating that the conserved replicative function of the FPC in stabilizing the replisome and facilitating fork progression is primarily responsible for preventing the fragility of chromosomes [53]. Recently, the C-terminal region of TIM was shown to exhibit specific binding to guanine (G)-quadruplex DNA structures, which is necessary for processive and efficient replication through these DNA sequences [54]. The direct interaction of TIM with DDX11/ChlR1 (Chl1 in yeast), a superfamily 2 (SF2) ATP-dependent DEAH-box 5´-3´ DNA helicase, was observed, indicating that TIM may assist DDX11 in recognition of G-quadruplex DNA structure and stimulate its unwinding activity [55, 56].

Telomeres are unique DNA structures that are intrinsically difficult to replicate; the replisome encounters not only repetitive telomeric DNA sequences but also protein barriers such as the telomere-protective shelterin complex and telomerases. Interestingly, deletion of Swi1 in *S. pombe* or long-term TIM knockdown in human cells results in telomere shortening [57, 58]. While TIM deficiency may be sufficient to reduce replication over naked telomeric DNA and thus to delay replication timing, given that TIM interacts with TRF1 and TRF2 (Taz1 ortholog in *S. pombe*) of the shelterin complex, which are known as the Myb DNA-binding domain-containing proteins that mediate a sequence-specific contact with double-stranded telomeric DNA, TIM may promote fork pausing at TRF1/2-marked telomere sequences to regulate telomere replication besides its role in replisome progression through telomeric DNA repeat sequences [58]. Short G-rich telomeric sequence repeats are prone to folding into G-quadruplexes that serve as a barrier to replication, and TIM and its interaction with the DDX11 helicase may be particularly important for resolving such secondary structures to carry out efficient replication through telomeres [59].

### Sister chromatid cohesion

Sister chromatid cohesion (SCC) is critical for the connection of newly replicated DNA pairs in order to achieve proper orientation of chromosomes on mitotic (or meiotic) spindles and subsequent chromosome segregation [60]. The cohesin complex stably associates with DNA during replication and forms a large ring structure that embraces the sister chromatids. The cohesin complex initially binds to DNA in a dynamic fashion at the start of S-phase, becoming stably bound upon acetylation of the cohesin subunit structural maintenance of chromosome 3 (SMC3) by the ESCO1/2 (Eco1/Ctf7) acetyltransferase [61–63]. An earlier biochemical study in *Caenorhabditis elegans* revealed a physical interaction between SMC1 and TIM, which further demonstrated that TIM depletion results in defective meiotic chromosome cohesion and embryonic lethality [64]. In *S. cerevisiae*, both Tof1 and Csm3 were identified as synthetic lethal partners of Ctf4, a Pol α-binding protein in the FPC known for its requirement for efficient SCC establishment [65, 66]. A similar synthetic lethal relationship was observed in *S. pombe* between Swi1 and the replication factor C (RFC/Ctf18) complex, where inactivation of Ctf18 or Swi1-Swi3 resulted in defective centromere cohesion [67]. Furthermore, it was shown that immunodepletion of Tim, Tipin, or And-1, leads to aberrant SCC in *Xenopus* egg extracts; mechanistically, both Tipin and And-1 promote stable chromatin association of Pol α for SCC establishment and DNA replication [68, 69]. Notably, while knockdown of TIM or TIPIN also results in SCC defects in human cells, it appears that TIM depletion exhibits a more profound defect in comparison to TIPIN knockdown [70]. This indicates that TIM may work independently to promote SCC, presumably by directly interacting with SMC1 and SMC3 within the cohesin complex whereas TIPIN indirectly exerts its role by stabilizing TIM at the replication fork [29]. Intriguingly, the aforementioned direct interaction between TIM and DDX11 may be relevant in SCC establishment as well, where TIM and DDX11 work together to promote cohesin binding to the replication fork [55, 56]. That said, deficiency of DDX11 or Chl1 is known to cause SCC defects [71–73]. Additionally, mutations in human DDX11 are associated with a rare cohesinopathy hereditary disease known as Warsaw breakage syndrome (WABS) [74]. These results suggest that the concerted interaction of the FPC with the SMC subunits and DDX11 may help stably anchor the cohesin complex to the replisome during DNA synthesis. Additionally, given that the MCM2-7 complex is known to recruit ESCO2 to a replication fork to promote SMC3 acetylation, TIM in the FPC may directly regulate the activity of the cohesin complex [75]. Collectively, these studies indicate that TIM coordinates DNA synthesis and SCC establishment at DNA replication forks, thus promoting chromosome stability and effective chromosomal segregation.

### Association with PARP1 at DNA replication forks

Poly(ADP-ribosyl)ation, catalyzed by the poly(ADP-ribose) polymerase (PARP) protein family, plays a multifaceted role in the DNA damage response and repair [76]. Upon DNA damage, PARP-catalyzed PAR conjugation onto target proteins as well as PARP itself, recruits downstream effector proteins to sites of DNA breaks to control multiple DNA–protein transactions, including basic excision repair, homology-directed repair, non-homologous end joining, and chromatin remodeling [77]. Intriguingly, recent studies have revealed additional roles of PARP1 at DNA replication forks, which include Okazaki fragment processing during lagging-strand synthesis, restriction of replication fork velocity, regulation of stalled fork remodeling and protection, and suppression of replication-associated daughter-strand DNA gaps [78–81]. Furthermore, association of PARP1 with its stimulatory factor, coactivator-associated arginine methyltransferase 1 (CARM1), was shown to determine the choice between fork remodeling versus DNA damage tolerance at stalled replication forks [82]. Interestingly, the C-terminus of TIM directly interacts with PARP1, but not with other PARP family member proteins, and PARP1 binding is required for the recruitment of TIM to laser-induced micro-irradiated DNA lesions to promote homology-directed repair [83, 84]. While the physiological relevance of this interaction remains unclear, given that PARP1 is involved in DNA replication process, it is tempting to speculate that the TIM-PARP1 interaction may play distinct roles in replication fork progression and stress responses at both ongoing and stalled forks. For instance, TIM contains both the G-quadruplex-binding and PARP1-interacting regions at its C-terminus, and PARP1 itself is known to recognize G-quadruplex DNA structures, which is associated with enzymatic activation of PARP1, thus raising the possibility that TIM may promote recognition and resolution of G-quadruplexes by recruiting PARP1 to them during replisome progression [54, 83, 85–87]. Constitutive activity of PARP1 has been noted during S-phase as a backup pathway for the detection and processing of unligated Okazaki fragments, suggesting that PARP1 may exert its function as part of the replisome associated with the FPC via TIM interaction [78, 88]. Characterizing the interplay between TIM and PARP1 at replication forks would be an important future direction to pursue to better appreciate the concerted roles of PARP1 in controlling both DNA replication and responses to replication-associated DNA damage.

## Roles of TIMELESS for the protection of stalled forks

### Regulation of the ATR–CHK1 replication checkpoint

**Fig. 3:**
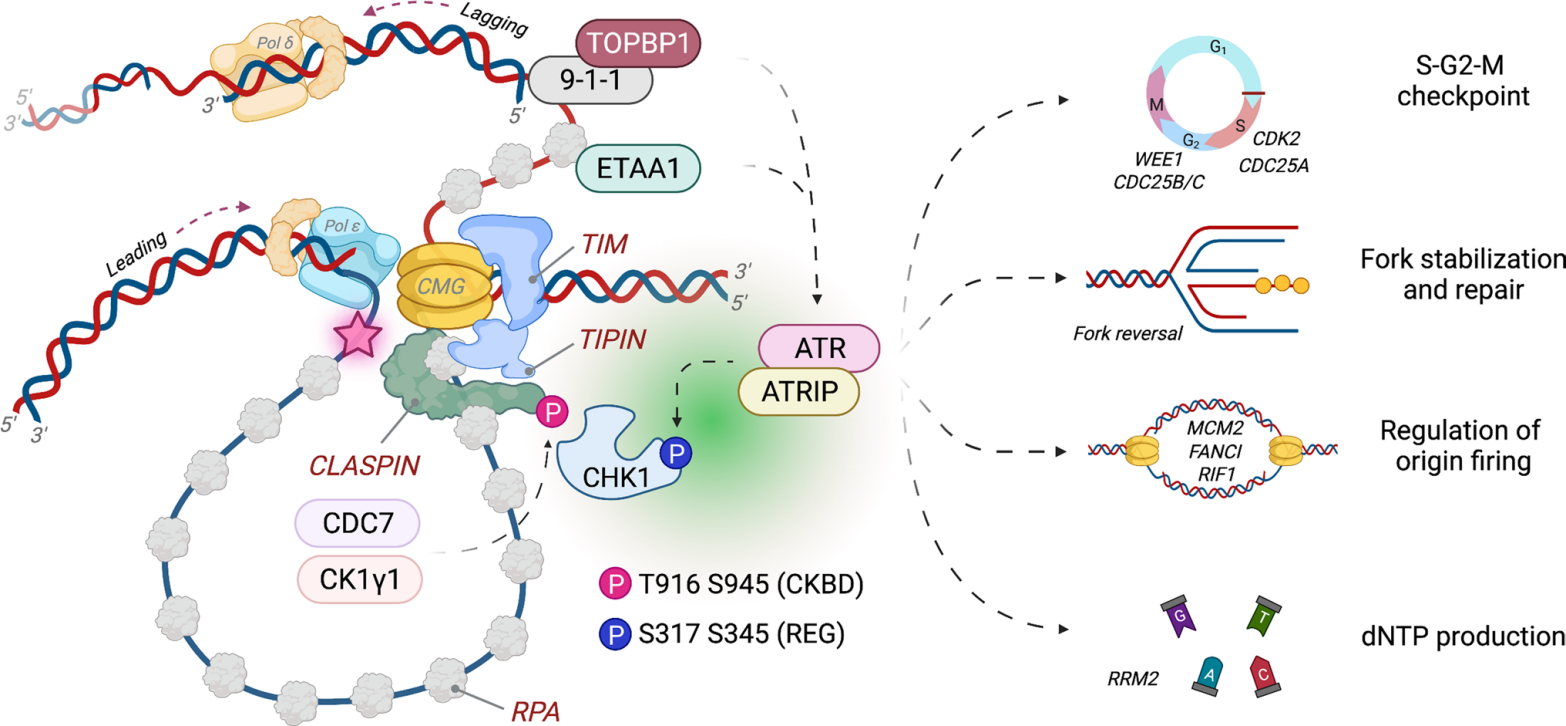
Regulation of the ATR–CHK1 checkpoint by the fork protection complex. One crucial mechanism of stalled fork protection mediated by the fork protection complex is to promote activation of the ATR–CHK1 replication checkpoint. Fork stalling at sites of DNA damage (marked by a red star) results in uncoupling of the CMG helicase and polymerase activities, generating a large stretch of ssDNA that becomes coated by RPA. ATR is recruited to RPA-bound ssDNA with the help of its targeting subunit ATRIP. TOPBP1 directly binds and activates ATR at the 5´ dsDNA–ssDNA junctions via its interaction with the RAD9–RAD1–HUS1 (9-1-1) complex, which itself is loaded by RAD17–RFC in an RPA-dependent manner. ATR is also activated by ETAA1 that directly associates with RPA. ATR phosphorylates CHK1 at Ser317 and Ser345 at the regulatory domain (REG) to activate CHK1 by releasing it from its autoinhibitory conformation. TIPIN in the TIM–TIPIN complex directly interacts with the RPA32 subunit of the RPA complex to help localize CLASPIN at stalled forks. CLASPIN is phosphorylated by CDC7 and CK1γ1 at Thr916 and Ser945 within a CHK1-binding domain (CKBD), which recruits CHK1 to stalled forks in close proximity with ATR to facilitate CHK1 activation by ATR. While ATR can phosphorylate many of its own targets by itself, it requires CLASPIN, TIPIN, and TIM as an adaptor complex to achieve efficient CHK1 phosphorylation. Accordingly, deficiency of TIM, TIPIN, or CLASPIN is sufficient for impairing ATR-dependent CHK1 phosphorylation under a variety of replication damage. Active CHK1 is dispersed from stalled forks, and in conjunction with ATR kinase activity, it collectively relays diverse checkpoint signaling pathways to protect the integrity of stalled replication forks and coordinate cell cycle transition. These include regulation of the S-G2-M cell cycle checkpoint to delay entry into mitosis, control of fork reversal and remodeling associated with fork repair, regulation of origin firing to activate local origins to continue replication while suppressing unnecessary dormant origin firing via phosphorylating multiple targets such as MCM subunits, FANCI, and RIF1, and stimulation of deoxynucleotide (dNTP) production (e.g., increase in the transcription of ribonucleotide reductase regulatory subunit 2 (*RRM2*))

The ATR kinase is a master regulator of DNA replication stress that coordinates multiple branches of cell cycle transition and fork stabilization [89]. ATR is recruited to ssDNA coated by RPA (replication protein A) at stalled replication forks and allosterically activated by TOPBP1 (DNA topoisomerase II β-binding protein 1) specifically in the presence of 5´-ended ssDNA–dsDNA junctions, or by ETAA1 (Ewing’s tumor-associated antigen 1), which is directly recruited to stretches of RPA-bound ssDNA [90, 91]. ATR in complex with ATRIP (ATR-interacting protein) phosphorylates many downstream targets, which collectively engage a number of pathways such as fork remodeling and repair, regulation of origin firing, dNTP production, and cell cycle control to protect the integrity of stalled forks and facilitate fork recovery [92]. Specifically, ATR-dependent CHK1 phosphorylation enforces the intra-S and G2-M checkpoints to coordinate cell cycle transitions by restricting CDK2, DDK, and Treslin activities during S-phase and preventing CDK1 activity via inhibitory activation of WEE1 and CDC25 degradation to delay mitotic entry [93]. Thus, regulation of CHK1 activation is a key mechanism in propagating ATR-dependent replication stress response signaling, and the FPC is well known for regulating this process (Fig. 3).

An earlier synthetic lethal screen identified Tof1, whose deficiency compromises Mec1 (ATR)-dependent Rad53 (CHK1) phosphorylation when combined with disruption of the DNA damage sensor Rad9, in conferring synergistic sensitivity to replication stress, including HU, ultraviolet (UV), and methyl methanesulfonate (MMS) [94]. Similarly, Swi1 mutants fail to activate Rad53-like checkpoint kinase Cds1, leading to irreversible fork collapse [36]. Multiple studies in human cells have also revealed that knocking down TIM or TIPIN leads to a defect in CHK1 phosphorylation in response to HU or UV treatment [2, 6, 33, 95, 96]. Furthermore, *Xenopus* egg extracts depleted of TIPIN showed defects in CHK1 phosphorylation when challenged with aphidicolin, an inhibitor of DNA replication [68]. Mechanistically, TIPIN directly interacts with the RPA2 subunit of the RPA complex via a conserved motif at its C-terminal half, and disruption of this interaction is sufficient to impair damage-induced CHK1 phosphorylation; this indicates that the RPA-TIPIN interaction is essential for localizing the FPC at stalled forks and facilitating the ATR-CHK1 replication checkpoint [33, 95]. Specifically, TIPIN recruits CLASPIN/Mrc1 to the replication fork; initially discovered as a CHK1-interacting protein, CLASPIN is known for stimulating ATR-dependent CHK1 phosphorylation in *Xenopus* egg extracts, in human cells, and in vitro [97–101]. This indicates that TIM–TIPIN acts as a platform to enrich ATR and CLASPIN localization at the RPA-ssDNA complex for enabling efficient CHK1 phosphorylation by ATR at stalled forks. The interaction between CLASPIN and CHK1 is mediated by the kinase domain of CHK1 which recognizes the phosphorylated CHK1-binding domain (CKBD) of CLASPIN [102, 103]. CLASPIN is phosphorylated by CDC7 and CK1γ1 at Thr916 and Ser945 within a CKBD, and is also regulated in an ATR-dependent manner, which contributes to recruiting inactive CHK1 to ATR at stalled forks [104, 105]. Additionally, ATR-dependent AND-1 phosphorylation enhances the CLASPIN-CHK1 interaction at ssDNA, further potentiating ATR-dependent CHK1 activation [106]. CHK1 binding to CLASPIN does not impact its kinase activity, indicating that CLASPIN mainly plays a structural role in promoting the recruitment of CHK1 such that it is in close proximity with ATR at stalled forks [107]. Furthermore, a cryo-EM structural study revealed a dynamic formation and dissociation of TIM–TIPIN onto ssDNA, which is regulated by distinct ssDNA length-dependent RPA conformations, suggesting that RPA may control the recruitment of the FPC to ssDNA-exposed stalled forks to orchestrate the engagement of proteins involved in fork stabilization and checkpoint activation in response to DNA replication stress [108]. Interestingly, TIPIN-depleted cells exhibit much higher levels of resistance to UV-induced inhibition of fork progression in comparison to TIM knockdown, indicating that TIPIN may exert a more profound role in checkpoint activation via its role in directly engaging the ATR checkpoint [33]. In yeast, Mec1-dependent Mrc1 phosphorylation at SQ/TQ cluster domains is also required for Rad53 activation, indicating that posttranslational modifications of the FPC may amplify the ATR–CHK1 signaling once enriched at stalled forks [109]. Such an intra-S checkpoint protein complex may be formed distinctly from the TIM–TIPIN heterodimer that couples the CMG and polymerases at active forks, depending on the extent of ssDNA exposed upon fork stalling. Whether TIPIN mainly acts as a stabilizing partner of TIM or exerts an independent role via its direct association with RPA has yet to be determined.

### Regulation of the replisome activity

Several studies argue against the notion that ATR signaling directly stabilizes the association of the replisome to replication forks under conditions of replication damage to prevent fork collapse [110, 111]. Nevertheless, phosphorylation of the FPC by the ATR and CHK1 signaling has been known to control replisome function at stalled forks to maintain fork stability and facilitate recovery. Rad53 deficiency generates excessive ssDNA in response to HU damage due to the uncoupling of leading- and lagging-strand DNA synthesis, indicating that the ATR-CHK1 checkpoint negatively regulates replisome progression at stalled forks [112]. Intriguingly, while the phosphorylation-defective Mrc1 mutant phenocopies the Rad53 mutant in its inability to counteract this asymmetric fork uncoupling, deletion of Mrc1 or Tof1, thus loss of proteins per se, rescues Rad53 deficiency and suppresses fork uncoupling, indicating the replication-promoting function of the FPC is unrestricted at stalled forks when it becomes refractory to the ATR–CHK1 checkpoint [113]. Similarly, Mrc1 phosphorylation by Rad53 slows down replication fork elongation in vitro [114]. Therefore, FPC-dependent replication pausing needs to be under the control of ATR and CHK1 to antagonize CMG unwinding at stalled forks; otherwise, unrestrained fork progression and subsequent replisome uncoupling would most likely sensitize a replication fork to accumulate extensive ssDNA and lead to fork collapse. In this sense, TIM ahead of the CMG may act as a negative regulator of fork progression under replication damage. Notably, inhibition of CMG activity by Rad53 that occurs independent of Mrc1 phosphorylation has been observed, indicating that Rad53 may be able to target other constituents of the replisome, perhaps the components within the CMG or the TIM–TIPIN complex [115]. Dynamic changes of TIM association in the replisome also control DNA replication. For instance, TIM is discharged from the replisome in response to reactive oxygen species caused by dNTP imbalance, which results in slowing down replication fork progression, indicating that the FPC modulates replisome architecture to adapt to metabolic stress [34].

The TIM–TIPIN complex is also necessary for efficient replisome disassembly during DNA replication termination. The CMG tightly associates with replication forks during elongation, which requires an active process of disassembling the replicative helicases for replication termination, specifically triggered by polyubiquitination of the MCM7 subunit of the CMG [116]. At the end of DNA replication, the *C. elegans* TIM–TIPIN stimulates the CMG helicase ubiquitination by promoting stable association of the CUL2^LRR1^ ubiquitin E3 ligase with the CMG, which in turn facilitates the disassembly of ubiquitinated CMG by CDC48 (p97 AAA^+^ unfoldase) and its adaptor UBXN3 (the orthologue of FAF1), further highlighting the role of TIM in controlling DNA replication processes by directly regulating replisome activity [117]. This active proteolytic process is distinct from the SCF^Pof3^-dependent degradation of the replisome components that is observed in the absence of Swi1, which may be engaged as part of the replication stress response to inactivate the replisome and thus slow down fork elongation [118].

### Stalled fork processing and protection

Stalled forks frequently undergo fork reversal, a process that involves regression of a stalled fork into a four-way junction to stabilize the damaged fork by promoting DNA damage tolerance and act as an intermediate for the repair and restart of a stalled fork [119]. The RAD51 recombinase promotes reversal of a stalled fork with the help of translocases such as ZRANB3, SMARCAL1, and HLTF, after which formation of RAD51 nucleofilaments protects the regressed arm from nucleolytic degradation [120, 121]. This RAD51 loading is stimulated by BRCA2, which prevents excessive fork degradation by the MRE11 nuclease [122, 123]. Accordingly, in pathological conditions where BRCA2 function is defective, the unprotected regressed arm is subjected to nuclease-mediated degradation [124, 125]. Recent DNA fiber analysis demonstrated that depletion of TIM causes excessive resection of HU-induced stalled forks, which is rescued by the loss of SMARCAL1 or MRE11 activity, indicating that TIM is required for the protection of reversed forks from nucleolytic degradation [2]. The MCM8/9 AAA^+^ helicase complex, known to be involved in replication-associated HR repair, also plays a multifunctional role in switching from supporting normal replisome progression to promoting RAD51 loading and suppressing the enzymatic processing of stalled forks [126]. A similar role of AND-1 in counteracting MRE11-dependent stalled fork processing and suppressing ssDNA gap formation was noted in avian DT40 cells [127]. This function requires the WD40 repeat domain, but not the HMG DNA-binding motif of AND-1, indicating that interactions with key protein factors, presumably with HR factors, may be crucial for promoting stalled fork protection. While the exact mechanism through which the FPC is involved in stalled fork protection remains unclear, dynamic remodeling of stalled forks by the FPC may be necessary for establishing a DNA repair intermediate that is favorable for the accommodation of factors involved in fork protection, repair, and recovery, including BRCA1/2 and RAD51. The iPOND (isolation of protein on nascent DNA) analysis has revealed that TIM and CLASPIN progressively dissociate from HU-induced stalled forks, suggesting that disengagement of the replisome may be an important step for fork reversal [110]. While displacement of TIM from the replisome in response to oxidative stress has been noted, whether it is the initiation of the signaling cascade culminating to active fork remodeling or just a passive way of slowing down fork progression remains to be determined [34]. How changes in replisome architecture under replication damage, perhaps controlled by multiple ATR- or CHK1-dependent posttranslational modifications of the FPC and the replication machinery, influence fork protection and recovery would be an important future direction for research.

## TIMELESS in cancer and therapeutic application

Given its essential role in genome maintenance, loss of TIM function is expected to promote genome instability and tumorigenesis. In this sense, it is intriguing to observe that TIM is upregulated in a variety of cancers and this high expression is significantly associated with the advanced tumor stages and poor prognosis [128–133]. It appears that TIM is involved in tumor development and progression through regulation of multiple oncogenic pathways. For instance, TIM promotes breast cancer cell growth by enhancing the synthesis of sphingosine-1-phosphate (S1P), an essential biomolecule for sphingolipid metabolism and mitochondrial respiration [134]. TIM overexpression also augments the self-renewal capacity of breast cancer stem cells by upregulating the transcriptional activity of MYC [128]. In addition, TIM functions as an estrogen receptor α (ERα) coactivator to stimulate ERα-induced gene expression and breast cancer cell growth [135]. Increased expression of TIM has been shown to promote ovarian cancer cell growth by exerting a wide influence on immune cell infiltration and activation, especially on tumor-associated macrophages [136]. That said, TIM is upregulated in various ways, including E2F1- and E2F4-mediated transcriptional activation, promoter hypomethylation, and ERK activation [129, 133, 137].

**Fig. 4:**
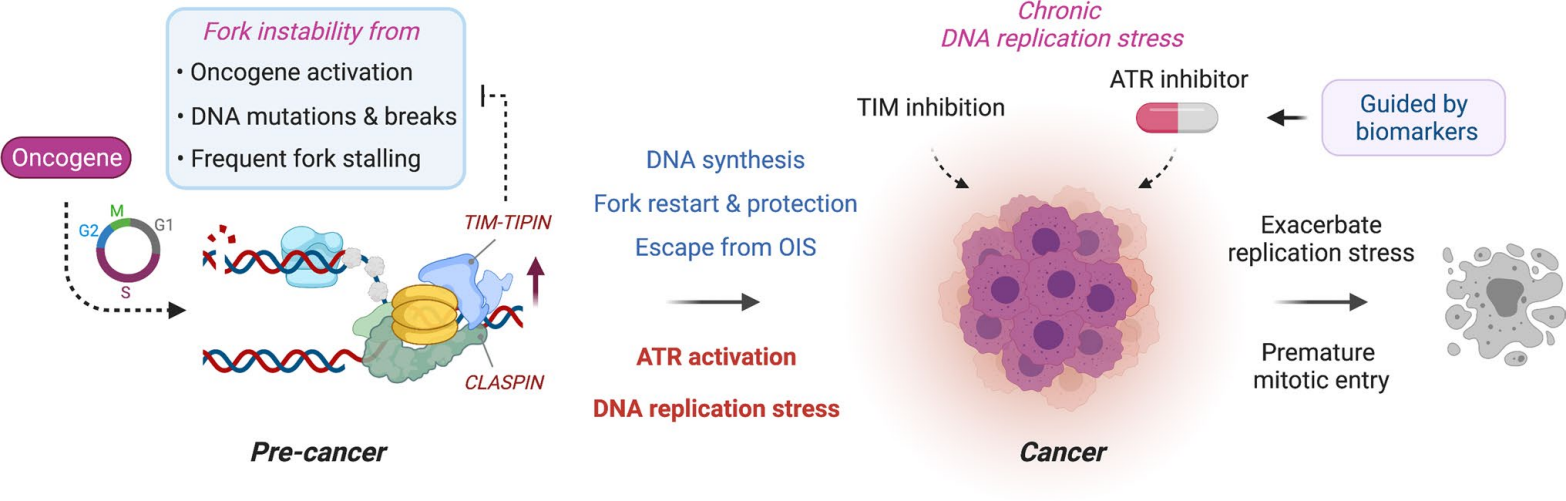
The oncogenic potential of TIM, a target for cancer therapy. Proliferating cells in the early step of tumorigenesis suffer compromised DNA replication fork stability due to a variety of genotoxic stress including oncogene activation, DNA mutation and breakage, and frequent fork stalling originating from a defect in DNA replication and repair. Increased expression and activity of the fork protection complex, including TIM and CLAPSIN, enables cells to tolerate elevated DNA replication stress by facilitating DNA synthesis, fork protection and restart, and completion of DNA replication in time. Upregulation of fork stabilization activity may also overcome oncogene-induced senescence (OIS), paving a way to oncogenic transformation. Progression from this precancerous status in turn triggers the ATR checkpoint to counteract the increasing amount of replication stress. Subsequently, persistent DNA replication stress and upregulated activity of TIM and ATR become a vulnerability point of cancer cells for therapeutic intervention. Increased expression of TIM is observed in a variety of cancers. Accordingly, hyper-reliance on the fork protection mechanism and checkpoints predicts that modulating TIM levels or disrupting the TIM interaction with other replication fork-associated factors may sensitize cells to ATR inhibition, which together exacerbates DNA replication stress and forces premature mitotic entry, resulting in a catastrophic failure of cancer cells. Differential expression of the fork protection complex and associated regulatory proteins could also be developed as biomarkers to help predict disease progression and therapeutic responses to replication stress-inducing agents

Together, these data suggest that high expression of TIM may be selected as an advantage for tumor development and progression (Fig. 4). Enhanced TIM activity at DNA replication forks may be necessary for efficient replisome progression in cells with precancerous lesions and for counteracting chronically high levels of DNA replication stress due to the inherent hyper-proliferative nature of cancer cells. The specific role of TIM in protecting stalled forks from degradation may also be needed for maintaining fork stability under frequent replication damage. Subsequently, this may confer a survival advantage for cancer cells with extensive genomic rearrangements, copy-number variations, and deregulation of the DNA damage response pathways [130]. Indeed, high levels of TIM and CLASPIN have been shown to increase a tolerance to oncogene-induced replication stress by protecting the stalled replication forks of cancer cells [138]. These cancer cells are expected to exhibit a vulnerability while they handle high levels of DNA replication stress, and exacerbating this replication stress using ATR inhibitors, or other checkpoint inhibitors that target CHK1 or WEE1, is thought to be a promising strategy for cancer therapy [139]. Accordingly, destabilizing TIM or disrupting the essential interactions of TIM at replication forks may provide a useful way to specifically target and kill cancer cells that have adapted to oncogene-induced replication stress or in part sensitize cancer cells to checkpoint inhibitors or cytotoxic chemotherapy. Indeed, knocking down TIM leads to a hyper-reliance on ATR activity to counteract the replication stress that originates from replisome uncoupling and ssDNA accumulation, suggesting that targeting ATR could synergize with replisome dysfunction [140]. Additionally, TIM expression could be utilized as a diagnostic and prognostic biomarker to stratify patients for better staging of disease progression and determination of treatment strategies. For instance, there is a positive correlation between high TIM levels and the aggressiveness and poor patient survival observed in low-grade non-small cell lung cancer (NSCLC), alluding to a therapeutic value of TIM during the surgical resection and subsequent adjuvant therapy of this disease [132, 138].

## Concluding remarks and future directions

In this review, we have highlighted the versatile roles of TIM, as part of the FPC within the replisome, in coordinating various DNA replication processes and the stress response under conditions of replication damage. As a scaffold that shapes the overall structure of the replication fork, we envision that its function is truly dynamic, switching back and forth between fork progression, fork pausing, and fork protection, thus adapting to new DNA–protein environments encountered during replisome progression. The action of TIM must be coordinated with other components of the FPC, i.e., TIPIN, CLASPIN, and AND-1, which exert distinct structural and functional roles during DNA replication and checkpoint signaling, as well as with additional replication fork-associated factors such as SDE2, DDX11, and PARP1. Several conceptual and technical advances have been accomplished, including biochemical and structural determination of the FPC for replisome control, new insight into the regulation of the FPC by the replication checkpoint, and elucidation of new roles of TIM in stalled fork protection. Built upon these observations, there are several outstanding questions that await further research. First, the molecular mechanisms by which TIM manages multiple DNA replication and repair processes have yet to be determined. These include its intersection with DNA damage tolerance, fork reversal, and repriming processes that influence fork progression and velocity, and a comprehensive understanding of the protein interactomes with TIM would be highly beneficial for understanding how choice of distinct pathways is determined at replication forks. Second, whether TIM can function independently of the TIM–TIPIN complex remains elusive. A division of labor may exist in which additional TIM molecules augment distinct processes of DNA replication, while the RPA–TIPIN interaction may be specialized for the propagation of the replication stress response signaling. If so, how TIM stability could be maintained without TIPIN is an important question to ask. Third, posttranslational modifications of the FPC have not been well characterized, however they are expected to play a major role in the fork pausing and remodeling necessary for the protection of stalled forks. As exemplified by CLASPIN/Mrc1 regulation, the ATR-CHK1 checkpoint would be responsible for modifying components of the FPC to fine-tune its function and stability. Additional modifications, including ubiquitination, SUMOylation, and poly(ADP-ribosyl)ation, would certainly provide another layer to FPC regulation. Lastly, the oncogenic role of TIM in mitigating high levels of DNA replication stress is an exciting concept that could be highly applicable to cancer therapy. Understanding how TIM shapes a favorable environment for tumor development and maintenance would establish an important therapeutic basis toward targeting TIM activity as a way to sensitize cells to additional DNA replication stress. Ultimately, knowledge on the fundamental DNA replication mechanisms and the stress responses associated with the FPC will provide a foundation for the development of strategies that exploit inherent vulnerabilities of the DNA replication fork in cancer cells for therapeutic interventions.

## Data Availability

Data sharing not applicable to this article as no datasets were generated or analysed during the current study.
